# Molecular footprint of Medawar’s mutation accumulation process in mammalian aging

**DOI:** 10.1111/acel.12965

**Published:** 2019-05-06

**Authors:** Zeliha Gözde Turan, Poorya Parvizi, Handan Melike Dönertaş, Jenny Tung, Philipp Khaitovich, Mehmet Somel

**Affiliations:** ^1^ Department of Biological Sciences Middle East Technical University Ankara Turkey; ^2^ Usher Institute of Population Health Sciences and Informatics University of Edinburgh Edinburgh UK; ^3^ European Molecular Biology Laboratory, European Bioinformatics Institute EMBL‐EBI Wellcome Trust Genome Campus Cambridge UK; ^4^ Department of Evolutionary Anthropology Duke University Durham North Carolina; ^5^ Department of Biology Duke University Durham North Carolina; ^6^ Duke Population Research Institute Duke University Durham North Carolina; ^7^ Center for Neurobiology and Brain Restoration Skolkovo Institute of Science and Technology Moscow Russia; ^8^ CAS Key Laboratory of Computational Biology, CAS‐MPG Partner Institute for Computational Biology, Shanghai Institutes for Biological Sciences Chinese Academy of Sciences Shanghai China

**Keywords:** aging, antagonistic pleiotropy, evolution, gene expression, genetic drift, mutation accumulation, protein sequence conservation

## Abstract

Medawar's mutation accumulation hypothesis explains aging by the declining force of natural selection with age: Slightly deleterious germline mutations expressed in old age can drift to fixation and thereby lead to aging‐related phenotypes. Although widely cited, empirical evidence for this hypothesis has remained limited. Here, we test one of its predictions that genes relatively highly expressed in old adults should be under weaker purifying selection than genes relatively highly expressed in young adults. Combining 66 transcriptome datasets (including 16 tissues from five mammalian species) with sequence conservation estimates across mammals, here we report that the overall conservation level of expressed genes is lower at old age compared to young adulthood. This age‐related decrease in transcriptome conservation (ADICT) is systematically observed in diverse mammalian tissues, including the brain, liver, lung, and artery, but not in others, most notably in the muscle and heart. Where observed, ADICT is driven partly by poorly conserved genes being up‐regulated during aging. In general, the more often a gene is found up‐regulated with age among tissues and species, the lower its evolutionary conservation. Poorly conserved and up‐regulated genes have overlapping functional properties that include responses to age‐associated tissue damage, such as apoptosis and inflammation. Meanwhile, these genes do not appear to be under positive selection. Hence, genes contributing to old age phenotypes are found to harbor an excess of slightly deleterious alleles, at least in certain tissues. This supports the notion that genetic drift shapes aging in multicellular organisms, consistent with Medawar's mutation accumulation hypothesis.

## 1 INTRODUCTION

To date, more than 300 hypotheses have been postulated to explain senescence, that is, age‐related loss of function and increase in mortality rates (Medvedev, 1990). The mutation accumulation (MA) hypothesis, an evolutionary explanation for aging first developed by Rose (1991) and Medawar (1952), is among the simplest and most influential of such hypotheses. It states that negative selection will be inefficient against alleles that exhibit harmful effects only late after maturation. Such alleles can eventually fix through genetic drift and thus contribute to observed senescent phenotypes (Kirkwood & Austad, 2000). The MA hypothesis generates several testable predictions. For instance, (a) genetic variance in fitness‐related traits, such as reproductive success or survival, should increase with age (Flatt & Schmidt, 2009; Rose, 1991); (b) inbreeding depression should increase with age; (c) alleles associated with late‐onset disease should segregate at higher frequencies than early‐onset disease alleles; and (d) genes expressed at late age should be under weaker purifying selection and evolve faster in their sequence, than those expressed at young age. To date, a number of studies have reported empirical evidence broadly consistent with these predictions. Several studies have shown age‐related increase in genetic variance in fitness‐related traits in either laboratory populations (e.g., *Drosophila melanogaster:* refs. (Charlesworth & Hughes, 1996; Hughes, Alipaz, Drnevich, & Reynolds, 2002)) [but see refs. (Promislow, Tatar, Khazaeli, & Curtsinger, 1996; Shaw, Promislow, Tatar, Hughes, & Geyer, 1999)] or natural populations (e.g., Soay sheep and red deer: ref. (Wilson et al., 2007)). In hermaphroditic snails, in an indirect test of the expectation regarding inbreeding depression, outbreeding was reported to mitigate age‐related increase in mortality (Escobar, Jarne, Charmantier, & David, 2008). In humans, the heritability of CpG methylation patterns was shown to increase with age for about 100 genome‐wide loci (although here, possible fitness consequences were not evaluated) (Robins et al., 2017)⁠. Finally, studying >2,500 human genetic variants linked to 120 genetic diseases, Rodríguez et al. (2017)⁠ reported that variants associated with late‐onset disease tend to segregate at higher frequencies than those associated with early‐onset disease.

Beyond those cited above, few studies have used empirical data to test the MA hypothesis. In particular, the conceivably variable contribution of MA to the aging processes affecting different species and different tissues has not yet been comparatively evaluated. Furthermore, with the exception of a few extreme cases such as the CAG repeat variants in the *huntingtin* gene that cause Huntington's disease, we have limited understanding into the nature and prevalence of late‐expressed substitutions, a central element of MA (Flatt & Schmidt, 2009).

The role of MA in aging therefore awaits testing through new approaches that encompass a larger number of traits, a wider array of species, different tissues, and molecular data. One such approach would be to take advantage of widely available transcriptome data, in particular genome‐wide gene expression datasets that include adult individuals of varying age. Such transcriptome datasets have traditionally been used to identify functional processes affected by or underlying senescence, although they can also be used to test evolutionary theories, as we show here.

In previous work, we used prefrontal cortex transcriptome age‐series from humans to investigate whether protein sequence conservation varies among genes that are highly expressed at different ages (Somel et al., 2010). This analysis showed that relatively highly expressed genes in young versus old adults are evolutionarily more conserved than those relatively highly expressed genes in old versus young adults, which we call age‐related decrease in conservation of the transcriptome (ADICT). Although this observation appeared broadly consistent with the MA hypothesis, the work analyzed only one brain region and did not distinguish between two distinct processes: (a) up‐regulation of lowly conserved genes with age and (b) down‐regulation of highly conserved genes with age. Both processes could generate the ADICT effect, but only (a) would be predicted by MA.

Here, we expand our investigation to include five mammalian species and 16 different tissue types. First, we study the prevalence of the ADICT pattern across multiple mammalian aging datasets, using estimates of protein and regulatory sequence conservation across mammals. Second, we ask whether genes up‐regulated late in life show low evolutionary conservation, as predicted by MA. In other words, we test whether slightly deleterious mutations are more likely to fix in genes that are more highly expressed in old age, such as genes that respond to age‐associated tissue damage (López‐Otín, Blasco, Partridge, Serrano, & Kroemer, 2013; Salminen, Kaarniranta, & Kauppinen, 2012).

## 2 RESULTS

### 2.1 Age‐related decrease in conservation of the transcriptome

We collected published transcriptome age‐series of young and old adults of five mammalian species, generated using RNA‐sequencing or microarrays (*Homo sapiens*, *Macaca mulatta*, *Macaca fascicularis*, *Rattus norvegicus*, *Mus musculus; n* = 66 datasets and 2,461 unique samples in all). The datasets represent transcriptomes of different brain regions (humans, macaques**,** rats, and mice), muscle (humans, rats, and mice), artery (humans, macaques, and rats), skin (humans and mice), kidney (humans and mice), liver (humans and mice), and lung (humans and mice). Heart, adipose, adrenal gland, blood, colon, esophagus, thyroid, and uterus datasets were only available from humans and spleen only from mice. The analyzed datasets included variable sample sizes (*n* = 9–116 individuals, mean = 37.2), and human ages ranged from 16 to 106 years, macaque ages from 4 to 28 years, rat ages from 3 to 30 months, and mouse ages from 8 to 130 weeks (Table S1).

We first studied congruence in age‐related gene expression change across the 66 datasets. For this, for each gene in each dataset, we calculated the Spearman correlation coefficient between gene expression level and individual age (*ρ*EA). We then compared datasets to estimate pairwise similarity in *ρ*EA values across common genes. *ρ*EA values were mostly (71% of comparisons) positively correlated across datasets, indicating that the same genes’ expression levels were similarly affected by aging across tissues and species (Figure S1).

As a measure of gene sequence conservation, we used estimates of purifying selection on protein sequence (*ω*0), calculated by Kryuchkova‐Mostacci and Robinson‐Rechavi (2015) and estimated for the human or the mouse branch using the branch‐site model (Zhang, Nielsen, & Yang, 2005). *ω*0 is the *dN*/*dS* ratio calculated for those sites determined to be under purifying selection and thus is expected to be a direct measure of the strength of purifying selection on a gene. We further calculated an adjusted protein conservation metric (*ω*0*) for each gene, factoring out the possible effects of GC content, CDS length, intron length, intron number, mean expression, median expression, maximum expression, tissue specificity, network connectivity, phyletic age, and number of paralogs, using a multiple regression model following Kryuchkova‐Mostacci and Robinson‐Rechavi (2015). The value −*ω*0* (*ω*0* multiplied by −1) represents the main protein sequence conservation metric we used in our analysis, where more positive values represent more conserved genes. Note that −*ω*0* is expected to be more powerful for detecting negative selection than metrics using intraspecies variation (a larger number of events are being evaluated in interspecies comparisons) or simple *dN*/*dS* (sites predicted to be neutrally evolving are not included in −*ω*0*).

**Figure 1:**
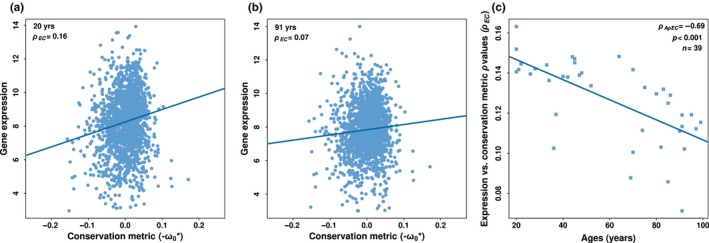
Relationship between gene expression level and protein conservation. Examples of gene expression level versus protein conservation metric correlations (a) for a 20‐year‐old human and (b) for a 91‐year‐old human, in the postcentral gyrus of the brain (data from Berchtold et al., 2008). The analysis includes only age‐related genes detected in this dataset (at *q* < 0.10). Each point represents a gene (*n* = 1688). The *x*‐axis shows the protein sequence conservation metric, where more positive values reflect higher conservation across mammals. The *y*‐axis shows log2‐transformed gene expression levels. The expression–conservation *ρ* values (*ρ*EC) are indicated in the inset. To improve visualization, we removed genes with disproportionately low conservation metrics (*n* = 3) in panels (a) and (b). Note that our correlation statistic, Spearman, is not affected by such potential outliers. (c) Age‐dependent change in expression–conservation *ρ* values in the human postcentral gyrus, based on age‐related genes in the same dataset as panels (a) and (b). The *y*‐axis shows expression–conservation *ρ* values (*ρ*EC) calculated for each individual in this dataset (*n* = 39). The *x*‐axis shows the ages of individuals. The *ρ* value between age and expression–conservation correlation (*ρ*AρEC) is indicated in the inset.

**Figure 2:**
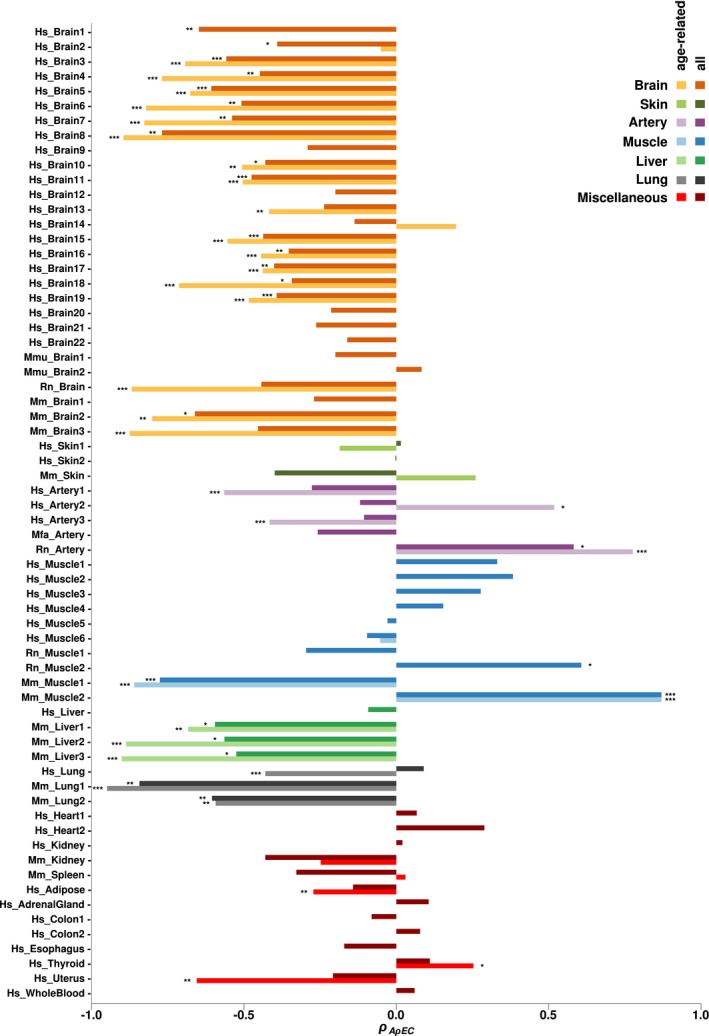
Age‐dependent changes in transcriptome conservation. The *x*‐axis shows the Spearman correlation coefficient (*ρ*AρEC) between individual age and expression–conservation correlations (*ρ*EC described in Figure 1). The statistics are calculated separately for each dataset, and for significant age‐related genes in that dataset (light bars), as well as for all expressed genes (dark bars). On the *y*‐axis, the species name (Hs: *Homo sapiens*, Mmu: *Macaca mulatta*, Mmf: *Macaca fascicularis*, Rn: *Rattus norvegicus*, Mm: *Mus musculus*) and tissue name are reported for each dataset. Note that in 26 of 66 datasets, where light bars are missing, significant age‐related genes could not be identified. The asterisks indicate nominal significance levels in the Spearman correlation test, (*): *p* ≤ 0.05, (**): *p* ≤ 0.01, (***): *p* ≤ 0.001. In the analysis using age‐related genes, all 28 datasets showing nominal significance for ADICT remained significant at *q* < 0.10 after applying Benjamini–Hochberg correction

We then investigated the prevalence of ADICT in mammalian aging. To do so, we first calculated the Spearman correlation coefficient between gene expression levels for each individual and the protein sequence conservation metric (which we call *ρ*EC) across all expressed genes (Figure 1a,b). Note that the conservation metric (−*ω*0*) is a constant value per gene, while gene expression levels will differ among individuals. In mammals, a weakly positive *ρ*EC, indicating that more highly expressed genes tend to be more conserved in their protein sequence, has been consistently observed in previous work (Kryuchkova‐Mostacci & Robinson‐Rechavi, 2015; Subramanian & Kumar, 2004; Warnefors & Kaessmann, 2013). The correlation suggests that a gene's expression level, among other factors, influences purifying selection pressure on its sequence, possibly as a consequence of selection against mistranslation and misfolding of highly expressed proteins (Drummond & Wilke, 2008; Pal, Papp, & Lercher, 2006). The magnitude of this correlation, though, can vary among individuals depending on their age, as genes expressed in young adults may be subject to stronger selection than genes expressed in old adults. To test this idea, in each dataset, we determined the correlation between individual ages and *ρ*EC (*ρ*AρEC). Figure 1c provides an example of such a pattern in one brain aging dataset (Berchtold et al., 2008), and Figure 2 shows the results across all datasets. Nonparametric correlation analysis is appropriate here, as the relation between individual ages and *ρ*EC mainly follows a linear trajectory (Figure S9 and Table S3).

In each dataset, we used two gene sets for testing ADICT: (a) genes showing significant age‐related change in expression levels (at Spearman correlation test *q*‐value < 0.10) and (b) all expressed genes. We conducted analyses using all expressed genes in order to avoid a reduction in statistical power in datasets with low sample sizes and to determine whether patterns that hold for strongly age‐associated genes also apply across the entire transcriptome (Table S2). Note that we could identify a set of significant age‐related genes at *q* < 0.10 only among 42 datasets. The remaining were mainly smaller datasets, and for these, we only conducted the analysis using all expressed genes (see Methods).

In the brain, we identified ADICT among 18/19 brain datasets with age‐related genes, that is, *ρ*AρEC values were negative (significant at nominal *p* < 0.05 in 17 datasets). When repeating this analysis with all expressed genes, 27/28 brain datasets had negative *ρ*AρEC values (significant at nominal *p* < 0.05 in 16 datasets). Together, these results support a general trend of ADICT in the brain (Figure 2). We also found nominally significant negative *ρ*AρEC values in the majority of liver (3/4) and lung (3/3) datasets, and in 2/5 artery datasets. In contrast, we found no consistent ADICT pattern in other tissues where we had multiple representative datasets, most notably in muscle (*n* = 10 datasets, among which we identified only one nominally significant case), as well as in heart, skin, kidney, and colon. We also note that in 6/10 muscle datasets, we did not detect significant age‐related expression change in gene‐by‐gene analyses (Table S2). Finally, in tissues where we had only one representative dataset, adipose and uterus showed a nominally significant ADICT pattern, while adrenal gland, colon, esophagus, thyroid, and blood did not.

Overall, 50/66 of the tested datasets (76%) showed an ADICT trend across all their expressed genes. Moreover, 28/42 of the datasets (67%) showed significant ADICT signatures across age‐related genes after correction for multiple testing (*q* < 0.10) (Figure 2). The pattern was driven by mainly brain, liver, lung, and artery, with 25 of the 28 datasets belonging to one of these four tissues. We focus on these 25 datasets in the following analyses.

We first sought to determine the robustness of this result with respect to our protein‐coding sequence conservation metric. For this, we repeated the analysis (a) using *ω*0 values without applying multiple regression, (b) using *ω* values (i.e., raw *dN*/*dS* values) obtained from the Ensembl database for “one‐to‐one orthologs” between human–mouse, human–elephant, and human–cow, and (c) using the mean PhastCons score (a conservation measure based on the UCSC database 100‐way vertebrate alignment) per gene as conservation metric. We further tested whether ADICT holds when we exclude (d) putatively positively selected genes (with *ω* > 1 in our data), (f) immune system genes known to be generally fast‐evolving (Mikkelsen et al., 2005; Nielsen et al., 2005; Zhang et al., 2005)⁠, and (e) genes down‐regulated with age in each dataset (ranging from *n* = 1,086 to 6,717). In addition, to exclude the possibility that ADICT signals are driven by gene expression changes involving only few functional processes (e.g., highly conserved developmental genes being down‐regulated), we calculated *ρ*AρEC separately for genes in each of the largest GO Biological Process (BP) categories (*n* = 19, each with node size >1,000 annotated genes) (Figure S2). We repeatedly observed ADICT (negative *ρ*AρEC values) as a general trend across the same 25 brain, liver, lung, and artery datasets, irrespective of the metric used, the gene sets, and GO categories involved (Table S2, Figure S2). Overall, ADICT appears to be a consistent pattern in multiple mammalian tissues.

### 2.2 Up‐regulation with age predicts low conservation

We next investigated two nonexclusive processes that could lead to ADICT: (a) Genes that show age‐related up‐regulation could be lowly conserved, consistent with MA, or (b) genes that show age‐related down‐regulation could be highly conserved, relative to genes showing no change in expression. The latter scenario could occur if a set of highly conserved genes (e.g.*,* synaptic genes) are down‐regulated during the postnatal lifespan, as previously reported (Lu et al., 2004; Somel et al., 2010), but would not provide direct support for MA.

**Figure 3:**
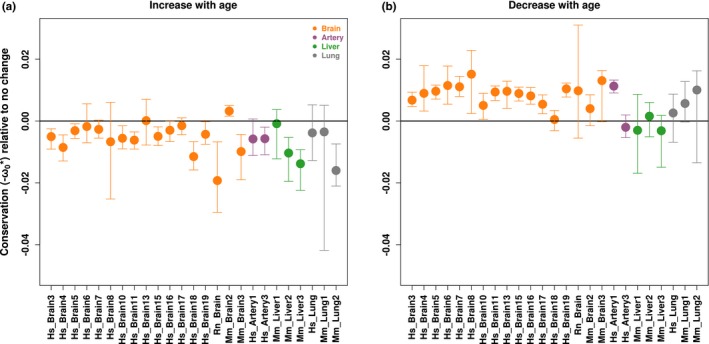
Mean conservation among gene sets with different patterns of age‐related change in expression levels. The plots show the mean conservation metric for genes that show age‐related increase (a) and age‐related decrease (b) in expression levels, compared to the mean conservation metric among genes that show no significant age‐related change in expression levels (see Methods). The error bars indicate 95% confidence intervals calculated by 1,000 bootstraps. The analysis includes the 25 brain, liver, lung, and artery datasets showing ADICT signatures

To test whether one or both of these scenarios underlie ADICT, we compared the mean conservation metric among (a) genes up‐regulated with age (*ρ*EA > 0.1, *q* < 0.1) and (b) genes down‐regulated with age (*ρ*EA < −0.1, *q* < 0.1), using (c) genes that show no age‐related changes in expression level as a control. We repeated this analysis across the 25 brain, liver, lung, and artery datasets showing the ADICT signature at *q* < 0.10, and using −*ω*0* as the conservation metric. We found results consistent with both scenarios (Figure 3): Genes down‐regulated with age were more strongly conserved than genes with no change (*n* = 22/25; 14 with bootstrap support >95%). Conversely, genes up‐regulated with age were more weakly conserved than genes with no change, in nearly all cases (*n* = 23/25; 15 with bootstrap support >95%). This is in line with the MA hypothesis: Genes that become more active late in life may be subject to stronger drift.

If age‐related up‐regulation is a general indicator of poor sequence conservation for a gene, the more tissues or the more species in which a gene shows age‐related up‐regulation, the less conserved it might be. To test this idea, we selected genes shared across the 25 ADICT datasets (in brain, liver, lung, and artery) and counted how many times each gene was up‐regulated with age. As predicted, we found a negative correlation between the number of datasets where a gene was up‐regulated with age and its conservation metric (*ρ* = −0.17, *p* < 0.001) (Figure S8a). Repeating this analysis for all 66 datasets also revealed a significant negative correlation (*ρ* = −0.23, *p* < 0.001) (Figure S8b). This suggests the presence of shared patterns of age‐related up‐regulation and low conservation across tissues and across species.

### 2.3 Functional analysis of ADICT

To find functionally coherent gene sets that may contribute to ADICT patterns in brain, liver, lung, and artery, we conducted Gene Ontology (GO) analysis for the three GO domains (BP, Cellular Component (CC), and Molecular Function (MF)). We separately analyzed (a) genes that showed increased expression with age and low conservation (IELC, consistent with MA) and (b) genes that showed decreased expression with age and high conservation (DEHC). For this, we ranked genes according to both expression‐age correlations (*ρ*EA) and the conservation metric (−*ω*0*), and investigated GO term enrichment in each of the 10% tails of the distributions. We sought shared GO categories enriched either in IELC genes or in DEHC genes across all the 25 brain, liver, lung, and artery datasets showing the ADICT signature. To determine the random expectation for shared GO categories, we randomly permuted ages of individuals in each dataset 1,000 times, calculated *ρ*EA again, and repeated the gene ranking and GO analysis.

IELC genes, which could be contributing to aging through the MA process, were enriched in the same 24 GO BP categories in all the 25 datasets (expected = 0; permutation test *p* < 0.001) (Figures S3 and S4, Table S4). These included categories related to apoptosis, inflammation, and the immune response, among others (see the REVIGO summary in Figure S4). In addition, four GO CC categories (expected = 0; *p* < 0.001) and one GO MF category (expected = 0; *p* = 0.022) were shared among IELC genes across the 25 datasets (Figure S3). Meanwhile, among DEHC genes, we found shared enrichment only in CC and MF categories (permutation test *p* < 0.05); significant gene sets included synapse‐ and signaling‐related functions (Figure S4) and Table S4).

### 2.4 Age‐dependent effects on regulatory region conservation

Finally, we asked whether ADICT extends to conservation of gene regulatory regions. To test this possibility, we calculated the mean PhastCons score per gene for (a) ±2000 bp around the transcription start site (TSS) and (b) the 3′‐UTR. We then repeated the ADICT analysis by substituting these two regulatory conservation metrics for −*ω*0*. This again revealed a heterogeneous trend toward ADICT across tissues, with consistent ADICT trends in brain, liver, and lung (Figures S5 and S6).

## 3 DISCUSSION

The MA hypothesis predicts that the burden of slightly deleterious germline substitutions will increase with age due to the declining force of negative selection, that is, due to the increasing influence of drift (Medawar, 1952). Our approach differs from earlier attempts to test this hypothesis (Charlesworth & Hughes, 1996; Escobar et al., 2008; Hughes, 2002; Promislow et al., 1996; Rodríguez et al., 2017; Shaw et al., 1999; Tatar, Promislow, Khazaeli, & Curtsinger, 1996; Wilson et al., 2007) in various respects. First, we used transcriptome data to study late‐expressed substitutions and age‐related phenotypic change. Second, instead of relying on intraspecies variation to estimate mutational load affecting a phenotype, we used interspecies divergence, which should be statistically more powerful as it involves a larger number of substitutions per gene. Third, we studied the mutational load in multiple tissues, thus considering the possibility that age‐dependent germline mutational load may vary among tissues, depending on tissue‐specific developmental patterns, mitotic capacity, damage accumulation, and consequences for organism‐level fitness.

We observed age‐related decrease in transcriptome conservation (ADICT) in datasets from brain, liver, lung, and artery, consistently across the mammalian species studied. Among datasets from all four of these tissues, genes up‐regulated with age showed low sequence conservation. Furthermore, across all datasets we studied, the frequency a gene was up‐regulated during aging inversely predicted its evolutionary conservation. These results are consistent with the MA hypothesis. In addition, processes that involve responses to aging‐related damage such as apoptosis and inflammation (Salminen et al., 2012) were enriched among genes that increased in expression during aging and had low conservation (IELC genes).

A number of questions remain. First, the methodology depends on mRNA expression data and whether all the observed aging‐related changes influence downstream, organism‐level phenotype is unclear. Further, if the function of a gene is modulated through other mechanisms, such as post‐translational modifications or alterations in the interaction partners, these will not be captured in our study.

Second, among the nine tissues for which we had >1 dataset, we could not systematically detect ADICT in the muscle, heart, kidney, skin, or colon. This observation is compatible with several distinct, nonmutually exclusive explanations. (a) Lack of an ADICT signal could represent false negatives due to experimental noise. The fact that the frequency of a gene's up‐regulation across all 66 datasets is negatively correlated with its conservation level (Figure S8b) supports this possibility. (b) ADICT propensity may vary due to differences in aging‐related expression change from tissue to tissue. This is most conspicuous in the comparison of brain and muscle, the two tissues with the richest data. For example, we consistently find a weaker signature of aging in muscle than in brain transcriptomes: We could only identify age‐associated genes in 4/10 muscle datasets compared to 18/19 of brain datasets. The functional properties of transcriptome changes also differ. For instance, while immune‐related genes are prominently up‐regulated during brain aging, the trend is significantly attenuated in muscle aging (Mann–Whitney *U* test *p* = 0.006; Figure S11). This difference is notable in view of the fact that immune‐related functions are enriched among IELC genes (Figure S4). This explanation does not, however, account for differences in the behavior of apoptosis‐related genes across tissues, where changes with age are similar in brain and muscle (Mann–Whitney *U* test *p* = 0.39). (c) Differences in ADICT propensity may also reflect differences in sensitivity to mistranslation errors among tissues: For example, neural tissue is highly sensitive to proteotoxicity and selection on protein sequence appears stronger on neuron‐related genes (Drummond & Wilke, 2008). Such differences among tissues, either in damage accumulation patterns, in the gene expression response to aging, or in tissue‐specific selection pressures, could influence the relative signal of MA in our analysis.

Third, shared IELC genes could represent genes evolving under positive selection instead of genes subject to drift. That is, low conservation might reflect the accumulation of beneficial substitutions (e.g.*,* in immune genes) rather than weakly deleterious ones. If we further assume that such genes’ up‐regulation is detrimental during aging, this scenario would be consistent with the antagonistic pleiotropy (AP) hypothesis, which argues that substitutions positively selected for their early life benefits may be harmful late in life (Williams, 1957). We find this unlikely, however, as (a) our main analysis is based on an estimate of negative selection rather than raw *ω*, and thus should not be affected by positive selection; (b) when we removed genes with *ω* > 1, or all immune‐related genes from our analysis, we still found the same ADICT and IELC signals (Figure S10); and (c) when we compared IELC genes and 370 genes identified to be under positive selection in humans through multiple genome scans (Cagan et al., 2016), we did not find more overlap than expected compared to the background set of all genes we analyzed (Fisher's exact test *p* = 0.3). Therefore, deficient purifying selection and accumulation of slightly deleterious substitutions by drift (Ohta, 2002), as predicted by the MA hypothesis, is a more parsimonious explanation for the observed IELC signal.

That said, our results do not exclude a role for AP in metazoan aging. Multiple aging‐related phenomena have been convincingly attributed to AP, such as negative correlations between early‐ and late‐life fitness in *Drosophila* (Sgrò & Partridge, 1999; Wit, Kristensen, Sarup, Frydenberg, & Loeschcke, 2013) and in humans (Carter & Nguyen, 2011; Rodríguez et al., 2017). In our previous work on aging brain transcriptomes, we had likewise interpreted the early initiation of synaptic gene down‐regulation as a case of runaway development possibly caused by an AP‐like process (Somel et al., 2010). In fact, the IELC phenomenon we describe here itself may partly be explained by AP, if some of the genes involved are affected by as‐yet undetected positive selection in early life. Aging is considered a highly heterogeneous phenotype shaped by multiple evolutionary and physiological processes, and joint roles for MA and AP in shaping aging‐related deleterious genetic load would be in line with this notion.

We do yet not understand how the IELC phenomenon might contribute to physiological decline in aging. Nevertheless, our finding that inflammation and apoptosis are shared functional characteristics of IELC genes in four tissues is telling, especially given the growing appreciation of the role of inflammaging, that is, low‐level inflammation observed in many aging tissues (Franceschi et al., 2000; López‐Otín et al., 2013; Salminen et al., 2012). There are multiple examples of how chronic inflammation can impair housekeeping functions, especially in the brain (e.g.*,* refs. (Salminen et al., 2012; Zhang et al., 2013)). It is also notable that the adipose dataset, a tissue with a known role in inflammaging (Mau & Yung, 2018), also shows an ADICT trend. Meanwhile, apoptosis is crucial for eliminating senescent cells during healthy aging and disruptions in apoptosis could lead to accumulation of dysfunctional cells over time. Conversely, apoptosis is also thought to have a role in neurodegenerative disease etiology, for example, in the case of Alzheimer's disease, by driving neuronal loss (Currais, Hortobágyi, & Soriano, 2009). Our results suggest that genes involved in cellular‐ and tissue‐level damage response, such as those with roles in inflammation and apoptosis, are subject to weaker purifying selection than other genes, possibly due to their relatively restricted recruitment early in life. The resulting mutational load may then lead to suboptimal regulation and function during aging in particular tissues, when these genes show elevated activity. The MA process may thus contribute to mammalian senescent phenotypes, although at varying levels in different tissues.

## 4 METHODS

### 4.1 Data preprocessing

We collected published mammalian transcriptome datasets that included young and old adults, preferentially with large sample sizes. We aimed to cover a diversity of tissues and to include multiple datasets per tissue if available, given conspicuous variation among transcriptome datasets in genome‐wide trends. Affymetrix.CEL files from 23 datasets (Barnes et al., 2011; Blalock et al., 2010; Edwards et al., 2007; Haustead et al., 2016; Jonker et al., 2013; Lee et al., 2011; Liu et al., 2013; Lu et al., 2004; Maycox et al., 2009; Miller et al., 2007; Misra et al., 2007; Niedernhofer et al., 2006; Qiu et al., 2007; Sinha et al., 2014; Somel et al., 2010; Swindell et al., 2012; Verbitsky et al., 2004; Welle, Brooks, Delehanty, Needler, & Thornton, 2003; Welle et al., 2004; Zahn et al., 2006) were downloaded from NCBI Gene Expression Omnibus (GEO) (Barrett et al., 2013) and from EBI Array Express (Kolesnikov et al., 2014) (Table S1). These raw datasets were preprocessed using the Bioconductor “affy” package “expresso” function (Gautier, Cope, Bolstad, & Irizarry, 2004). The selected options for the “expresso” function were as follows: “rma” for background correction, “quantiles” for normalization, and “medianpolish” for summarization; the procedure also includes log2 transformation (Bolstad, Irizarry, Astrand, & Speed, 2003). Whenever raw data were not available, the preprocessed series matrix files were downloaded from NCBI GEO; the datasets were log2‐transformed and quantile normalized if deemed necessary based on inspection of the downloaded data. RNA‐seq datasets were downloaded from genotype‐tissue expression (GTEx) (Ardlie et al., 2015). We chose 15 tissues from GTEx to represent a diversity of tissues. These datasets were processed using log2 transformation on the gene expression levels and quantile normalization using “preprocessCore” package in R (Bolstad et al., 2003). Preprocessing steps used on the analyzed datasets are presented in Table S1. Quantile normalization was preferred because the amount of gene expression level change that occurs during aging is known to be limited (Somel et al., 2010).

### 4.2 Probeset‐to‐gene conversion

Affymetrix probe set IDs were converted to Ensembl gene IDs using the Bioconductor “biomaRt” package (Durinck et al., 2005). We used the “useMart” function to select the dataset for the species of interest and the “getBM” function to retrieve the Ensembl gene IDs corresponding to Affymetrix probe set IDs. We then followed two steps: (a) If one probe set corresponded to more than one Ensembl gene, we removed that probe set and (b) if >1 probe set corresponded to one Ensembl gene, we chose the probe set which had the maximum expression value across all samples in that dataset. This approach used information only from the highest expressed and best‐measured transcript per gene in each dataset (in other words, we discarded information from more lowly expressed and possibly noisy transcripts in that dataset).

### 4.3 Age test and age‐related gene sets

In each dataset, genes showing age‐related change in expression levels were identified using the Spearman correlation test. We used the R “cor.test” function using the “method = ‘Spearman’” argument for calculating the age‐expression correlation coefficient *ρ*EA. The *p*‐values were corrected for multiple testing using the “p.adjust” function with the “Benjamini–Hochberg (BH)” method in R, yielding *q*‐values as a measure of the false discovery rate. We used the nonparametric Spearman rank correlation test to overcome several problems related to conducting meta‐analysis (e.g., expression levels in each dataset display unique and sometimes non‐normal distributions; outliers can influence the analysis). We used a *q*‐value cutoff of *q* < 0.10, which is a commonly used threshold (e.g.*,* refs. (Hartmann et al., 2009; Somel et al., 2010)). Among 66 datasets, 26 had a low number of age‐related genes (*n* < 50); therefore, to limit type II error, we did not include these datasets in analyses of age‐related gene sets. Gene set sizes for age‐related genes and all detected genes for all 66 datasets are shown in Table S2. Unsurprisingly, the number of age‐related genes is partially affected by sample size (at Spearman correlation test *ρ* = 0.35, *p* = 0.03), but this does not influence the main patterns we report with respect to ADICT (see below).

In each dataset, we further defined three gene sets based on the expression‐age Spearman correlation coefficient (*ρ*EA): (a) genes that showed age‐related increase, with *ρ*EA > 0.1 and *q* < 0.1; (b) genes that showed age‐related decrease, with *ρ*EA < −0.1 and *q* < 0.1; and (c) genes that show no change in expression level with age (*q* > 0.10). Here, in addition to the *q*‐value, we also used the correlation coefficient (*ρ*EA) as cutoff; this avoids including genes with small effect size that can be identified in large datasets (i.e., with high power) but not in small datasets. Genes with *q* < 0.10 and |*ρ*EA| < 0.1 were discarded from gene set‐based analyses.

### 4.4 ADICT

The ADICT pattern was calculated as the Spearman rank correlation between age and *ρ*EC in each dataset, (a) using age‐related genes, if detected in that dataset, and (b) using all genes in each dataset. The Spearman *p*‐values were corrected using the BH method as described above (across all datasets included in an analysis). We note that correlation between |*ρ*AρEC| and sample size across datasets was negative (*ρ*AρEC calculated for age‐related genes: *ρ* = −0.66, *p* < 0.001; *ρ*AρEC calculated for all genes: *ρ* = −0.47, *p* < 0.001). This is simply because finding large correlation coefficients is unlikely with large sample sizes. However, this pattern cannot explain why we observe a consistent trend for negative *ρ*AρEC values (i.e., ADICT) only in some tissues: For example, in the brain, 27/28 datasets show a negative *ρ*AρEC, whereas only 4/10 muscle datasets show a negative *ρ*AρEC.

### 4.5 Protein sequence conservation metrics

We used several types of metrics to estimate negative selection pressure on protein‐coding sequences.

First, we used *ω*0, a statistic based on coding sequence alignments across mammalian species. *ω*0 is estimated for the Homininae branch for human and the Murinae branch for mouse, using the branch‐site model (Zhang et al., 2005). In the branch‐site model, the branch of interest (the “foreground branch”) is permitted to have a different distribution of *dN*/*dS* values than the other branches in the phylogenetic tree (the “background” branches), which are constrained to have the same distribution of *dN*/*dS* value among sites. The branch‐site model thus estimates positive or negative selection pressure on a protein‐coding gene sequence. Here, we used the *dN*/*dS* ratio calculated for sites determined to be under negative selection. Thus, *ω*0 is expected to be a measure of the strength of negative selection on a gene. The values, calculated for each Ensembl gene, were downloaded from the Selectome database (Moretti et al., 2014).

This measure of *ω*0 can vary among genes due to multiple factors that are not the focus of this study. To disentangle the effects of such factors from the effect of protein sequence conservation per se, we used information on GC content, CDS length, intron length, intron number, mean expression, median expression, maximum expression, tissue specificity, network connectivity, phyletic age, and number of paralogs, which were directly obtained from the Supplemental Material of Kryuchkova‐Mostacci and Robinson‐Rechavi (2015). To remove the effect of these variables from *ω*0, we used the “lm” function in the R “stats” package to calculate the residuals (*ω*0*) from a multiple regression model with *ω*0 as the response variable and all other variables as predictors. The *ω*0* statistic was calculated separately for human and for mouse *ω*0 values. We used the human *ω*0* data in analyses involving primate transcriptome datasets and the mouse *ω*0* data in analyses involving rodent transcriptome datasets.

Second, we calculated conservation in protein‐coding regions between pairs of species separated by different evolutionary distances, using *dN* (nonsynonymous substitution rate) and *dS* (synonymous substitution rate) statistics downloaded from Ensembl Biomart (v.83) (Yates et al., 2016). Here, we used “one‐to‐one orthologs” between human–mouse, human–elephant, and human–cow, in order to identify whether evolutionary distance between species affects estimated levels of sequence conservation. Because *dN*/*dS* ratios measure the strength of both negative selection and positive selection, we repeated our analysis only using genes with *dN*/*dS* < 1 (i.e., excluding the genes most likely to evolve under recurrent positive selection). In addition, we used the R “biomaRt” package to select 3,171 genes assigned to GO categories and subcategories related to the immune system (“GO:0002376”), known to be fast‐evolving, and repeated the analysis after discarding these genes.

Third, we calculated the conservation of protein‐coding sequences using the PhastCons scores (phastcons100way) downloaded from the UCSC database (Siepel et al., 2005). Phastcons100way scores each base of the human genome based on the alignment of 99 vertebrate genomes to human. To find coding regions for each gene, we used the coding start and end positions from Ensembl Biomart (v.83), combining all isoforms per gene. We obtained a list of all PhastCons scores (phastcons100way) for the coding bases of each human gene via BEDTools (Quinlan & Hall, 2010) software and then calculated the mean PhastCons score value as a metric to represent conservation of that gene's coding region (Figure S7).

### 4.6 Regulatory region conservation metrics

To calculate conservation for 3′‐UTRs of mammalian genes, we first retrieved start and end positions of human gene 3′‐UTRs from Ensembl Biomart (v.83). Due to alternative splicing, one gene may be transcribed into multiple isoforms, leading to more than one 3′‐UTR per gene, which may overlap. Thus, for each gene, we selected all bases annotated as part of any isoform's 3′UTR. To calculate conservation levels of human gene promoter regions, we defined promoters as the 2,000 bp upstream and downstream of a gene TSS, which we again obtained from Ensembl Biomart (v.83). For genes with multiple TSSs, we selected all bases that were located in promoter regions. To overcome possible biases that may arise from the inclusion of conserved exon regions into the regulatory region boundaries, we discarded exonic regions within the 2,000 bp window around gene TSSs.

Using the BEDTools software package (Quinlan & Hall, 2010)⁠, we obtained a list of all PhastCons scores (phastcons100way) for the defined 3′UTR bases or promoter bases of each gene. We then calculated the mean PhastCons score value as a metric to represent that gene's 3′‐UTR or promoter region conservation.

### 4.7 Bootstrapping

Bootstrapping was performed using the “sample” function in R, with “replacement = TRUE.” We used bootstrapping to calculate 95% confidence intervals for the mean conservation metric among genes that showed (a) age‐related increases in expression levels, (b) age‐related decreases in expression levels, and (c) no age‐related changes in expression levels. For each case, we resampled genes 1,000 times and calculated the mean. To visually compare the conservation metric among datasets, we then subtracted the median for genes that showed no age‐related change, from genes that showed age‐related increase or age‐related decrease. The upper and lower 2.5% quantiles are plotted in Figure 3.

### 4.8 Testing linearity

To determine whether the relationship between individual age and *ρ*EC (calculated across age‐related genes) was linear across adulthood, we compared linear regression models and quadratic regression models for each dataset, with *ρ*EC as the response variable and age as the explanatory variable (using the R “lm” function).

### 4.9 Defining IELC and DEHC gene sets

We developed a nonparametric statistic, *z*, which simultaneously captures the relationship between a gene's expression and age, and the relative conservation level of a gene:

z=x2-y2,

where *x* is the rank of a gene's *ρ*EA (expression level vs. age correlation coefficient) across all detected genes in a dataset, and *y* is the rank of the same gene's conservation metric. Using squared values gives additional weight to differences between higher ranks. High values of *z* indicate genes that have relatively high expression and low conservation, whereas low values of *z* indicate genes that have relatively low expression and high conservation. After sorting *z* values, the top 10% of genes were included in the increasing expression and low conservation (IELC) gene set and the bottom 10% were included in the decreasing expression and high conservation (DEHC) gene set.

### 4.10 Gene Ontology analysis

Here, we sought to find functional groups associated with either IELC or DEHC patterns that were shared across datasets of a tissue and across all datasets. We conducted GO analyses for the three GO domains: BP, CC, and MF. For this, we (a) chose GO groups showing enrichment tendencies in each dataset, using liberal cutoffs (see below), (b) determined the overlap among chosen GO groups among datasets, and (c) tested the significance of the overlaps using random permutations of individual age in each dataset. Specifically, in each dataset, we chose GO groups with an odds ratio > 1, comparing either IELC or DEHC genes (the most extreme 10% tails of the *z* statistic's distribution described above) to the rest (90%). We preferred to use liberal odds ratio cutoff (>1) instead of a *p*‐value cutoff in order to avoid type II error and to ensure that datasets with different numbers of genes contributed equally to downstream analysis. We then counted the number of overlapping GO groups that were thus chosen (odds ratio > 1) across the 25 brain, liver, lung, and artery datasets showing the ADICT signature, or among different datasets for the same tissues. Next, we randomized ages of individuals in each dataset by conducting 1,000 permutations using the R “sample” function, calculated expression correlations with age, and repeated the GO analysis using these correlation values. We finally compared the number of GO groups that showed enrichment tendency (odds ratio > 1) in the random permutations, with the observed values.

In order to get GO annotations for genes, we used Ensembl biomaRt package in R. We propagated the annotations considering the GO hierarchy (downloaded using http://archive.geneontology.org/latest-termdb/, date of retrieval: June 17, 2015), so that GO terms include all genes that are associated with their descendent GO terms.

## CONFLICT OF INTEREST

None declared.

## AUTHORS' CONTRIBUTION

M.S. and P.K. conceived the study. M.S. supervised the study with contributions by J.T. and P.K. Z.G.T. and P.P. analyzed the data with contributions by H.M.D. and M.S. M.S. and Z.G.T. wrote the manuscript with contributions by P.P., J.T., H.M.D.
