# Supplementary material for: Molecular footprint of Medawar’s mutation accumulation process in mammalian aging
Source: Aging Cell. 2019 May 6;18(4):e12965. doi: 10.1111/acel.12965 (PMC6612638; doi:10.1111/acel.12965)
Supplement: Supplementary file 1 [file ACEL-18-e12965-s001.pdf]

Mutation accumulation differentially impacts aging in mammalian tissues

Zeliha Gözde Turan[1], Poorya Parvizi[1], Handan Melike Dönertaş[2], Jenny Tung[3], Philipp Khaitovich[4], Mehmet Somel[1]*

[1]Department of Biological Sciences, METU, Ankara, Turkey.
[2]European Molecular Biology Laboratory, European Bioinformatics Institute EMBL-EBI, Wellcome Trust Genome Campus, Hinxton, Cambridge, UK.
[3]Department of Evolutionary Anthropology, Duke University, Durham, NC, USA.
[4] CAS-MPG Partner Institute for Computational Biology, Shanghai, PRC.
*Corresponding author: msomel@metu.edu.tr

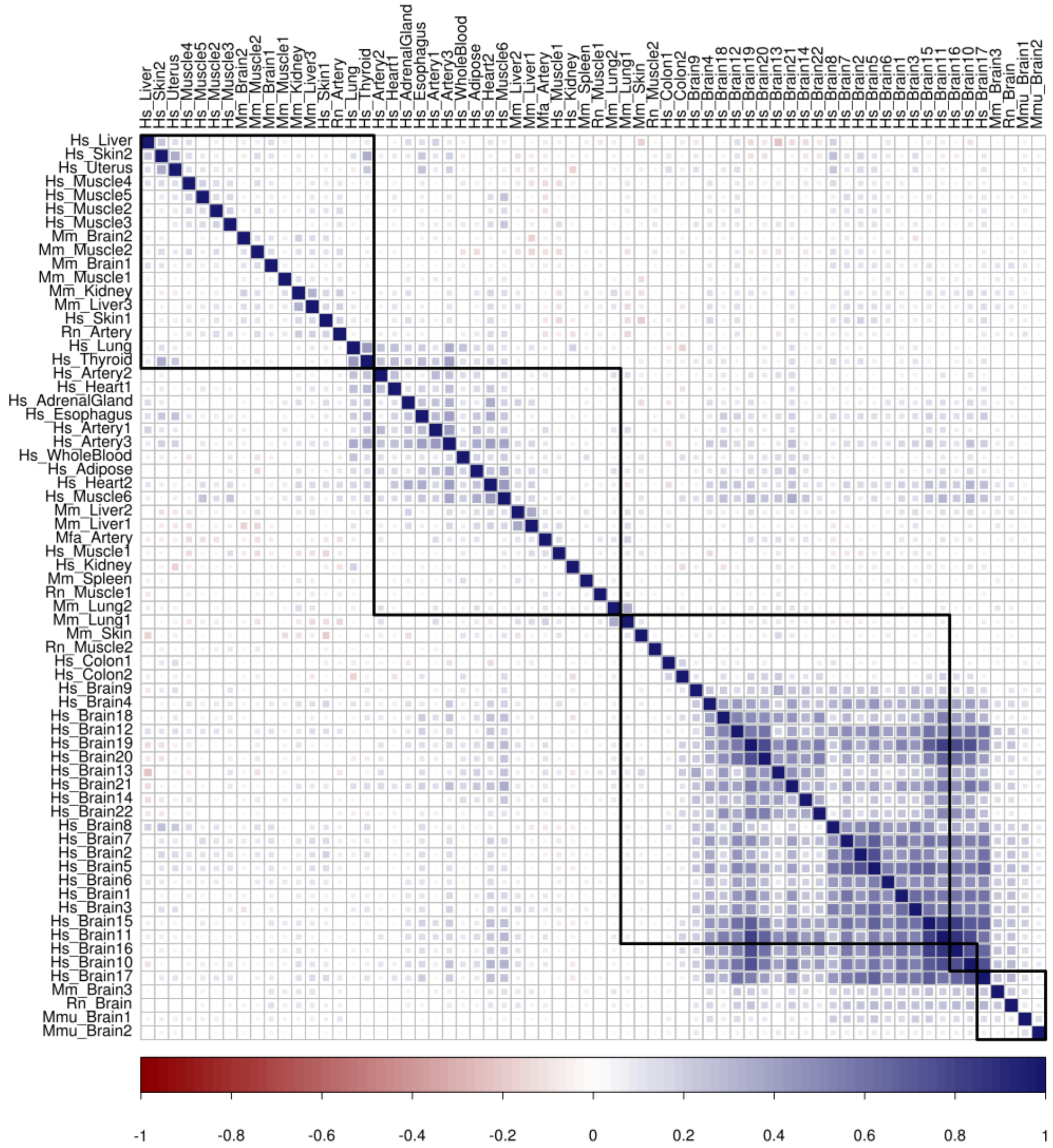

Supplementary Figure 1. Pairwise correlations of gene-level Spearman correlation coefficients between gene expression and age (ρ_{EA}), across all 66 datasets used in the analysis. Row and column names of the correlation matrix show each dataset, with the order determined by hierarchical clustering. Strong correlations are indicated with darker squares, red for negative and blue for positive. The number of common genes between any pair of datasets ranges from 2,387 to 21,323. The pairwise correlation coefficients across datasets range from -0.23 to 0.86. Overall there were positive correlations were found in 71% of 2211 pairwise comparisons. Among tissues belonging to the same dataset, 94% of pairwise comparisons were positively correlated among the 28 brain datasets, and 67% among the 10 muscle datasets. Among datasets belonging to the same platform, RNA sequencing and microarray datasets show 76% and 68% positive correlation among themselves, respectively.

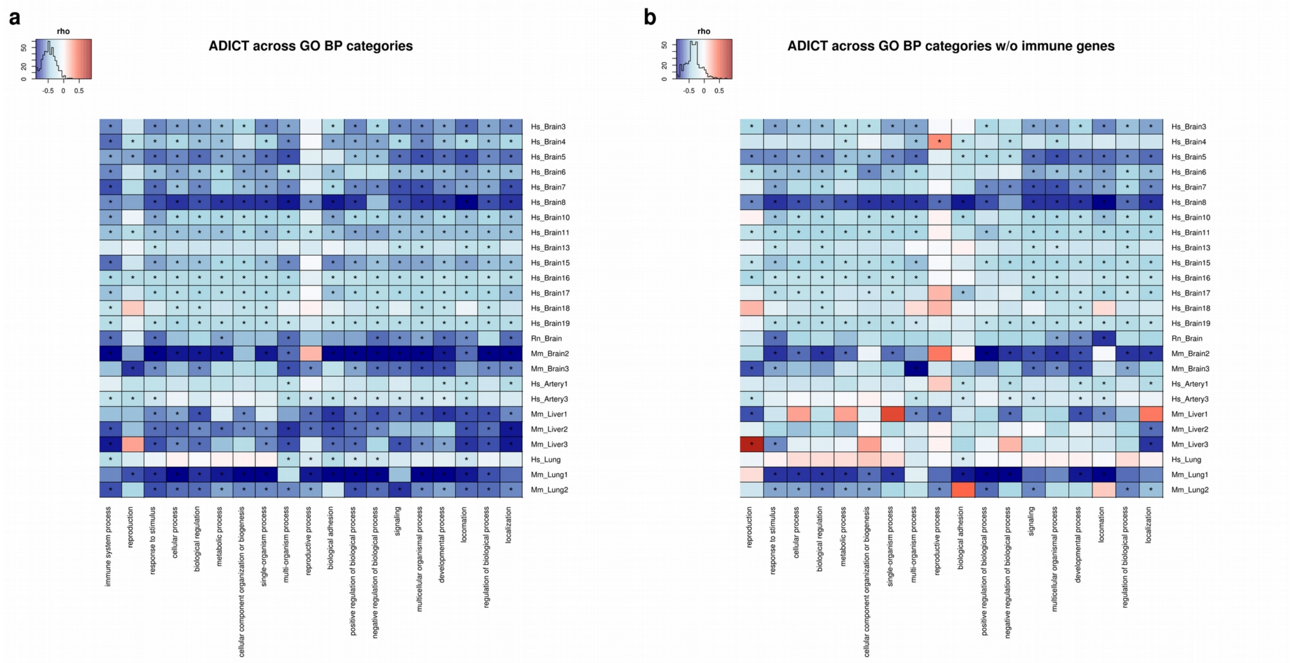

Supplementary Figure 2. Correlation between the conservation metric ($-\omega_0^*$) and expression level of genes categorized by GO BP categories that include (a) at least 1000 genes, and (b) at least 1000 genes and without immune system-related genes. These 19 GO BP categories were the only ones that had >1000 genes. Shared genes between categories were not excluded. Row and column names show each dataset and GO BP category respectively. Magnitude of Spearman correlation coefficient is indicated by the colour of the squares (darker colour shows stronger correlation): red for positive and blue for negative. The asterisks indicate, (*): $p \leq 0.05$. The analysis includes the 25 brain, liver, lung, and artery datasets showing ADICT signatures.

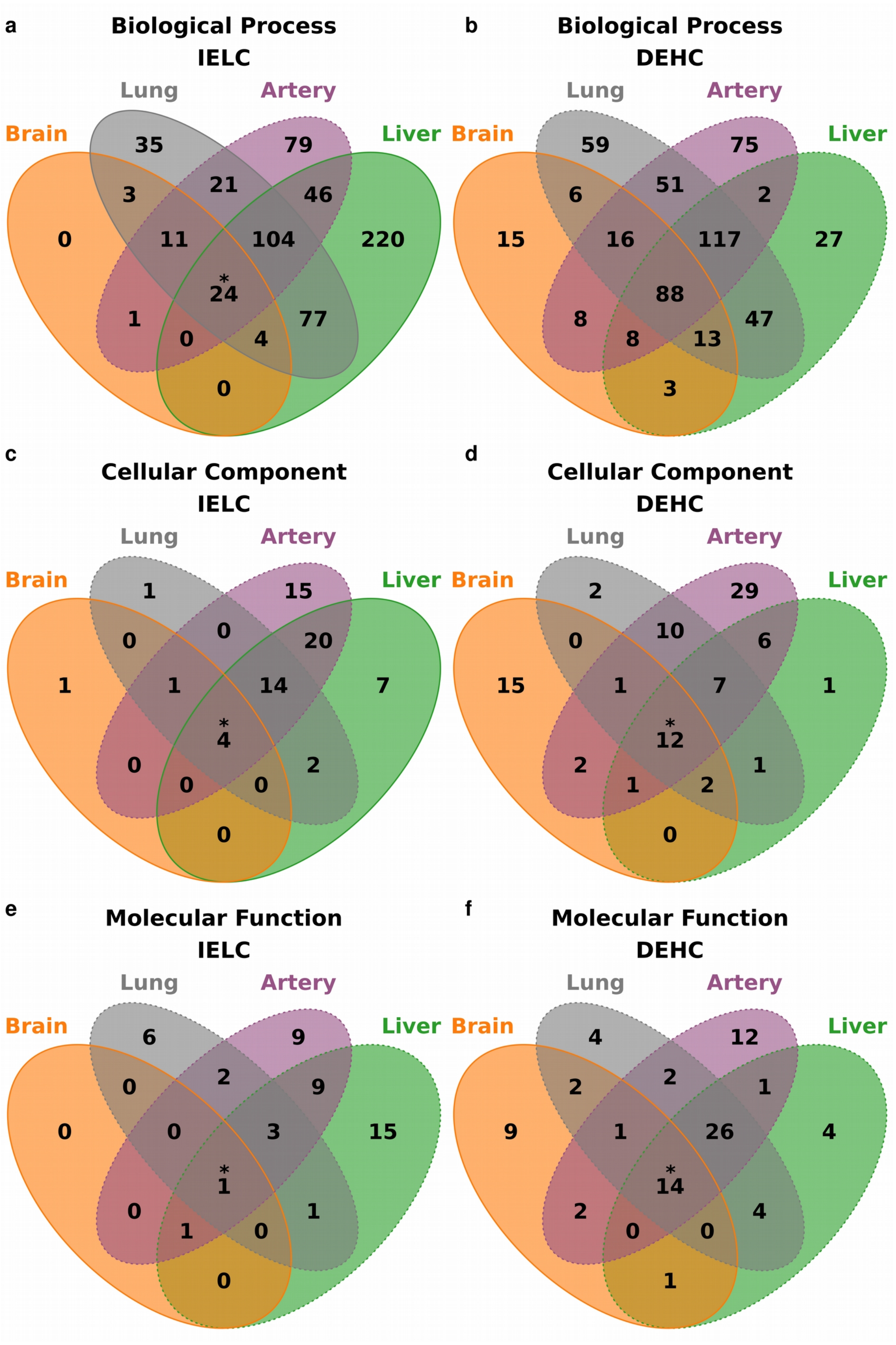

Supplementary Figure 3. Number of GO groups enriched for genes that show increased expression with age and low conservation (IELC), and for genes that show decreased expression with age and high conservation (DEHC) across different tissues. Panels a-f show GO group enrichment results for the three GO domains. Enrichment was calculated for each tissue and for

IELC or DEHC genes separately. If IELC (or DEHC) genes in a dataset showed overlap with a GO group with odds ratio > 1 (relative to other genes and other GO groups) and this was observed for all datasets of the same tissue (brain, n = 17; liver, n = 3; lung, n = 3; artery, n = 2 datasets showing ADICT), we assumed enrichment of IELC (or DEHC) genes in that GO group for that tissue (see Methods for an explanation of the rationale). The significance of the enrichment results was tested by random permutations at two levels: (a) categories found in each of the four tissues, and (b) for the categories shared between all four tissues (The asterisks indicate, (*): permutation test $p \leq 0.05$). In panels a-f, if a tissue is demarcated by a dashed line (*e.g.* artery and lung in panel a), this indicates lack of significant GO enrichment in datasets of that tissue ($p > 0.05$). **(a)** We found 24 GO Biological Process (BP) categories (expected = 0; permutation test $p < 0.001$) enriched for IELC across all 25 datasets. Supplementary Fig. 4 contains a summary of these results as provided by REVIGO (Supek et al. 2011). Among BP GO categories, n = 43 (expected = 2; permutation test $p < 0.001$), n = 475 (expected = 93; permutation test $p < 0.001$), and n =279 (expected = 85; permutation test $p = 0.002$) were enriched for IELC in brain, liver, and lung, respectively. For artery, we did not find a common significant enrichment for IELC based on the permutation test. **(b)** In the 17 brain datasets, 157 GO categories (expected = 112; $p = 0.028$) were enriched for DEHC. For other tissues, we did not find a common significant enrichment for DEHC based on the permutation test. **(c)** We found four GO Cellular Component (CC) categories (“lytic vacuole”, “lysosome”, “vacuole”, “extracellular space”) enriched for IELC across all 25 datasets (expected = 0; permutation test $p < 0.001$). Among CC GO categories, n = 6 (expected = 0; permutation test p = 0.005) and n = 47 (expected = 20; permutation test $p = 0.01$) were enriched for IELC in brain and lung, respectively. For artery and lung, we did not find a common significant enrichment for IELC based on the permutation test. **(d)** We found n = 12 GO Cellular Component categories enriched for DEHC across all 25 datasets (expected = 6; permutation test $p = 0.043$). Supplementary Fig. 4 contains a summary of these results. In the brain datasets, 33 GO categories (expected = 9; p < 0.001) were enriched for DEHC. For other tissues, we did not find a common significant enrichment for DEHC based on the permutation test. **(e)** We found n = 1 GO Molecular Function (MF) category (“protein homodimerization activity”) enriched for IELC across all 25 datasets (expected = 0; permutation test $p = 0.022$). In the brain datasets, n = 2 GO categories (expected = 0; $p = 0.05$) were enriched for IELC. For other tissues, we did not find a common significant enrichment for IELC based on the permutation test. **(f)** We found n = 14 GO Molecular Function (MF) categories enriched for DEHC across all 25 datasets (expected = 5; permutation test p = 0.006). Supplementary Fig. 4 contains a summary of these results. In the brain datasets, n = 29 GO categories (expected = 8; $p < 0.001$) were enriched for DEHC. For other tissues, we did not find a common significant enrichment for DEHC based on the permutation test.

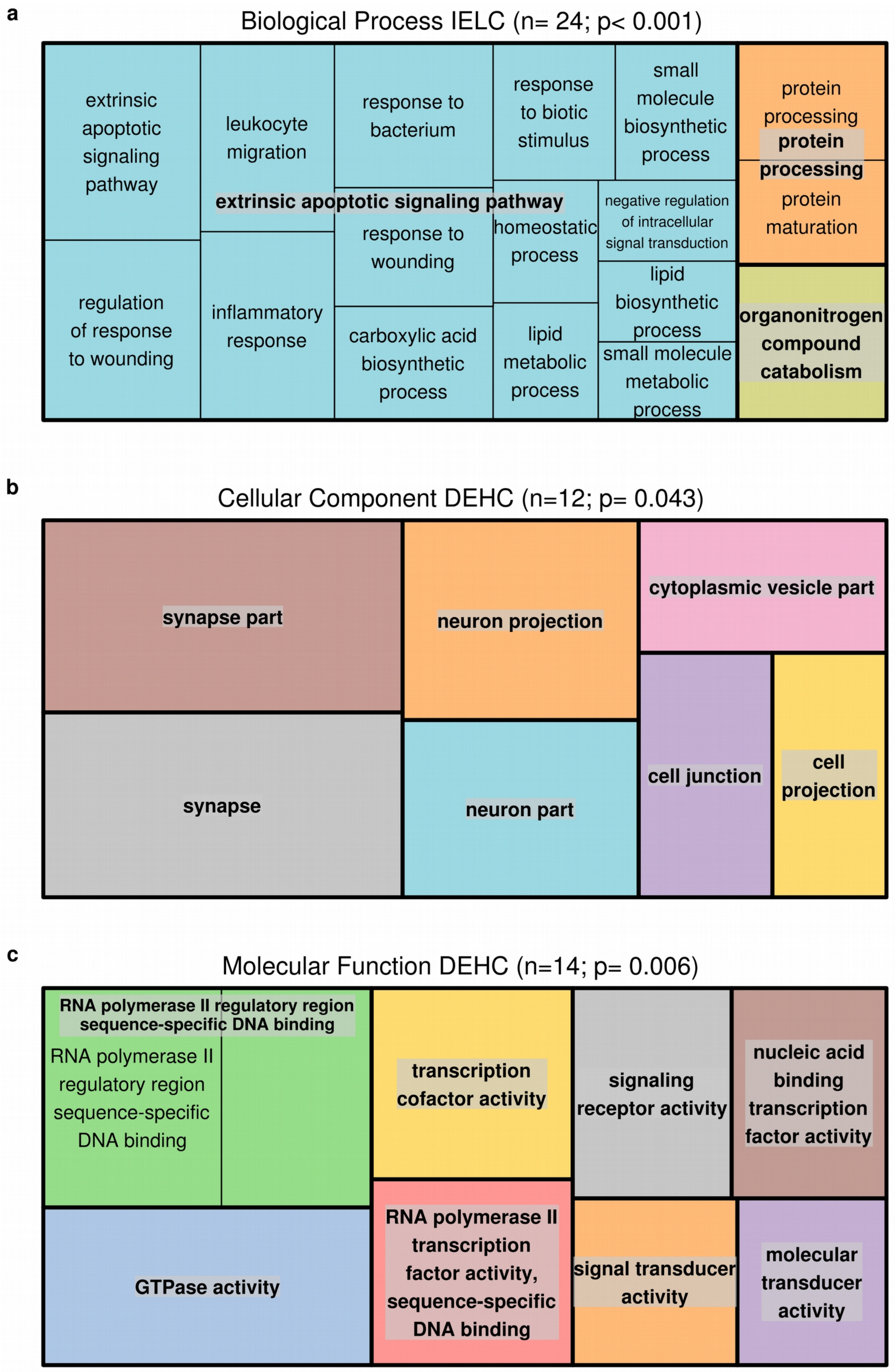

Supplementary Figure 4. Summary of GO groups shared in all 25 datasets showing ADICT, produced using the REVIGO software (Supek et al. 2011). GO categories were selected as enriched for IELH and DEHC genes, if they showed an odds ratio > 1 in each of the 25 datasets (relative to other genes in that dataset and genes in other GO categories). Clusters are shown by rectangles and

superclusters are separated by colour. **(a)** The 24 GO BP categories enriched for IELC across datasets. **(b)** The 12 GO CC categories enriched for DEHC across datasets. **(c)** The 14 GO MF categories enriched for DEHC across datasets (Supplementary Table 4).

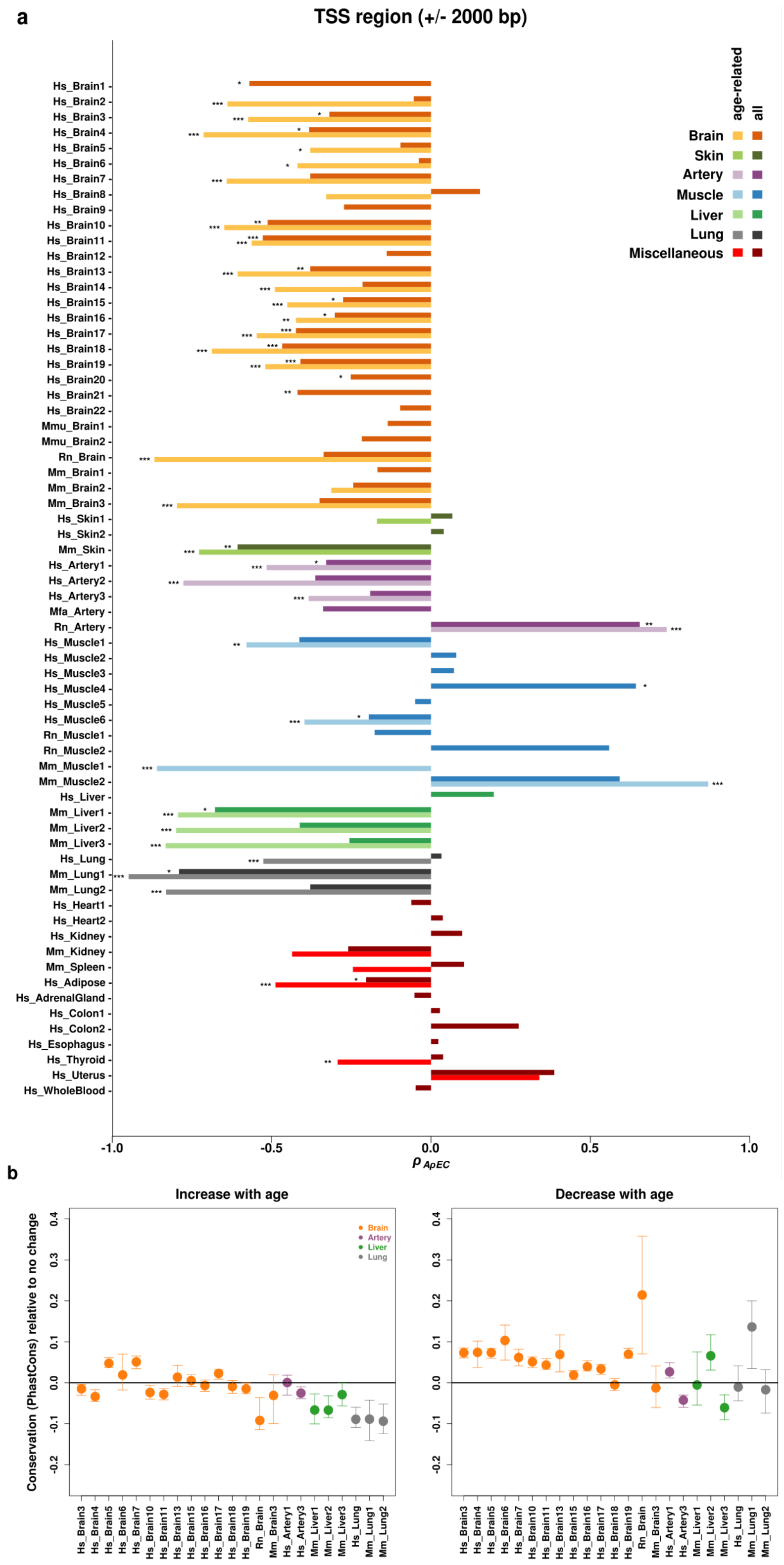

Supplementary Figure 5. Changes in the transcription start site (TSS) region (+/- 2000 bp) conservation (PhastCons) during aging. **(a)** The x-axis shows age-dependent change in expression level–regulatory region conservation correlation, measured by the Spearman correlation coefficient ρ. The results were calculated separately for each dataset, and for significant age-related genes in that dataset (light bars), as well as for all expressed genes (dark bars). Note that in 40 of 66 datasets

(cases where the light bar is missing), no significant age-related gene set could be identified at $q < 0.10$. The asterisks indicate, (*): $p \leq 0.05$, (**): $p \leq 0.01$, (***): $p \leq 0.001$. **(b)** Comparison of the conservation metric among gene sets showing different age-related expression level change patterns. The plots show mean conservation metric for genes showing age-related increase (left) and age-related decrease (right) in expression level, compared to mean conservation metric among genes showing no significant age-related change in expression level. The error bars indicate 95% confidence intervals calculated by 1,000 bootstraps. In 12 datasets (bootstrap support >95%), genes that show an increase in expression with age had lower regulatory region conservation, on average, than genes with no change. In 16 datasets (bootstrap support >95%) genes that show a decrease in expression with age also had higher conservation levels, on average, than genes with no change.

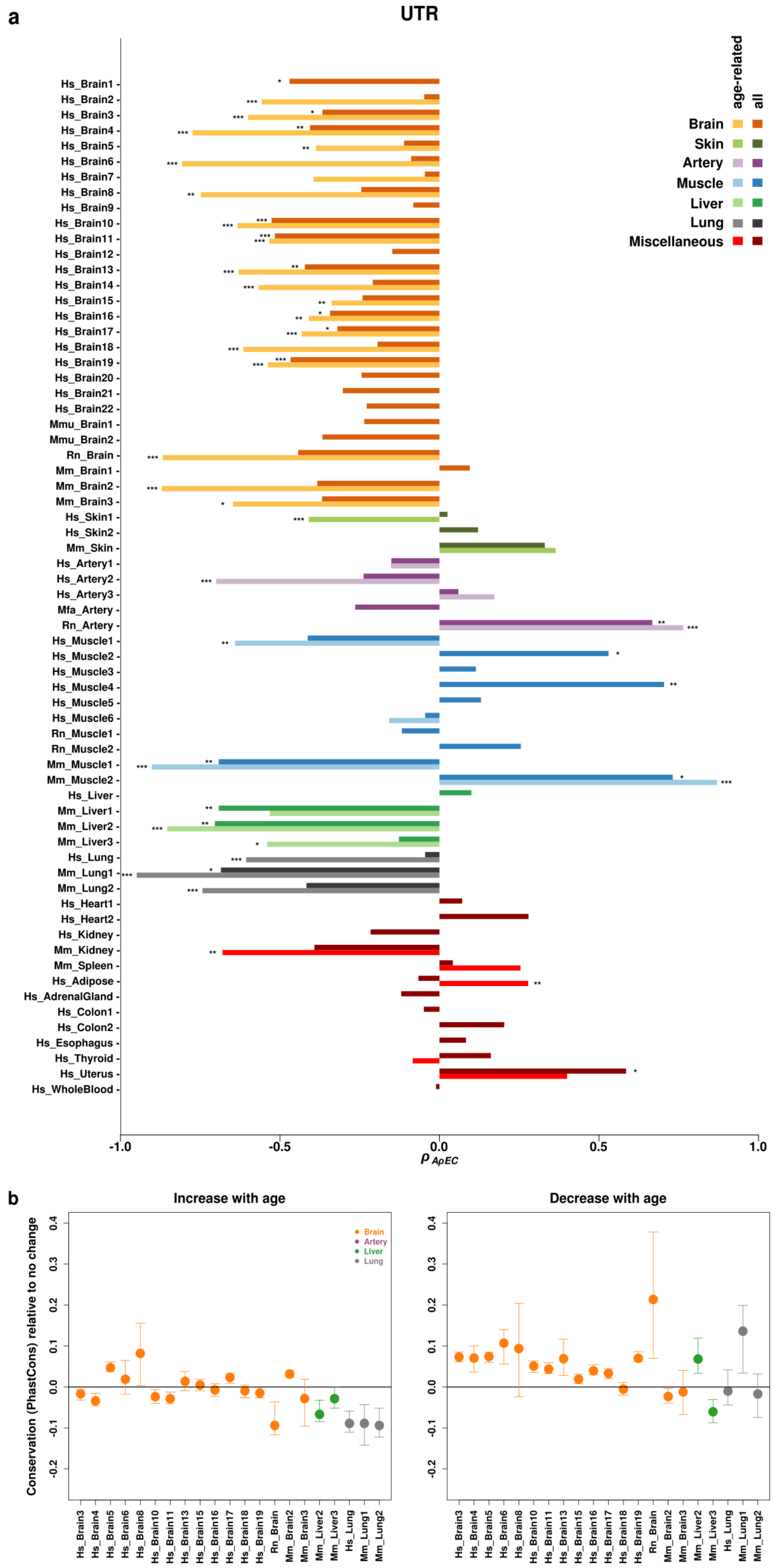

Supplementary Figure 6. Changes in 3’ untranslated region (3’-UTR) conservation (PhastCons) during aging. (a) The x-axis shows age-dependent change in expression level–regulatory region conservation correlation, measured by the Spearman correlation coefficient ρ. The results were calculated separately for each dataset, and for significant age-related genes in that dataset (light bars), as well as for all expressed genes (dark bars). Note that in 40 of 66 datasets (cases where the

light bar is missing), no significant age-related gene set could be identified at $q < 0.10$. The asterisks indicate, (*): $p \leq 0.05$, (**): $p \leq 0.01$, (***): $p \leq 0.001$. (b) Comparison of the conservation metric among gene sets showing different age-related expression level change patterns. The plots show mean conservation metric for genes showing age-related increase (left) and age-related decrease (right) in expression level, compared to mean conservation metric among genes showing no significant age-related change in expression level. The error bars indicate 95% confidence intervals calculated by 1,000 bootstraps. In 10 datasets (bootstrap support >95%), genes that show an increase in expression with age had lower regulatory region conservation, on average, than genes with no change. In 14 datasets (bootstrap support >95%) genes that show a decrease in expression with age also had higher conservation levels, on average, than genes with no change.

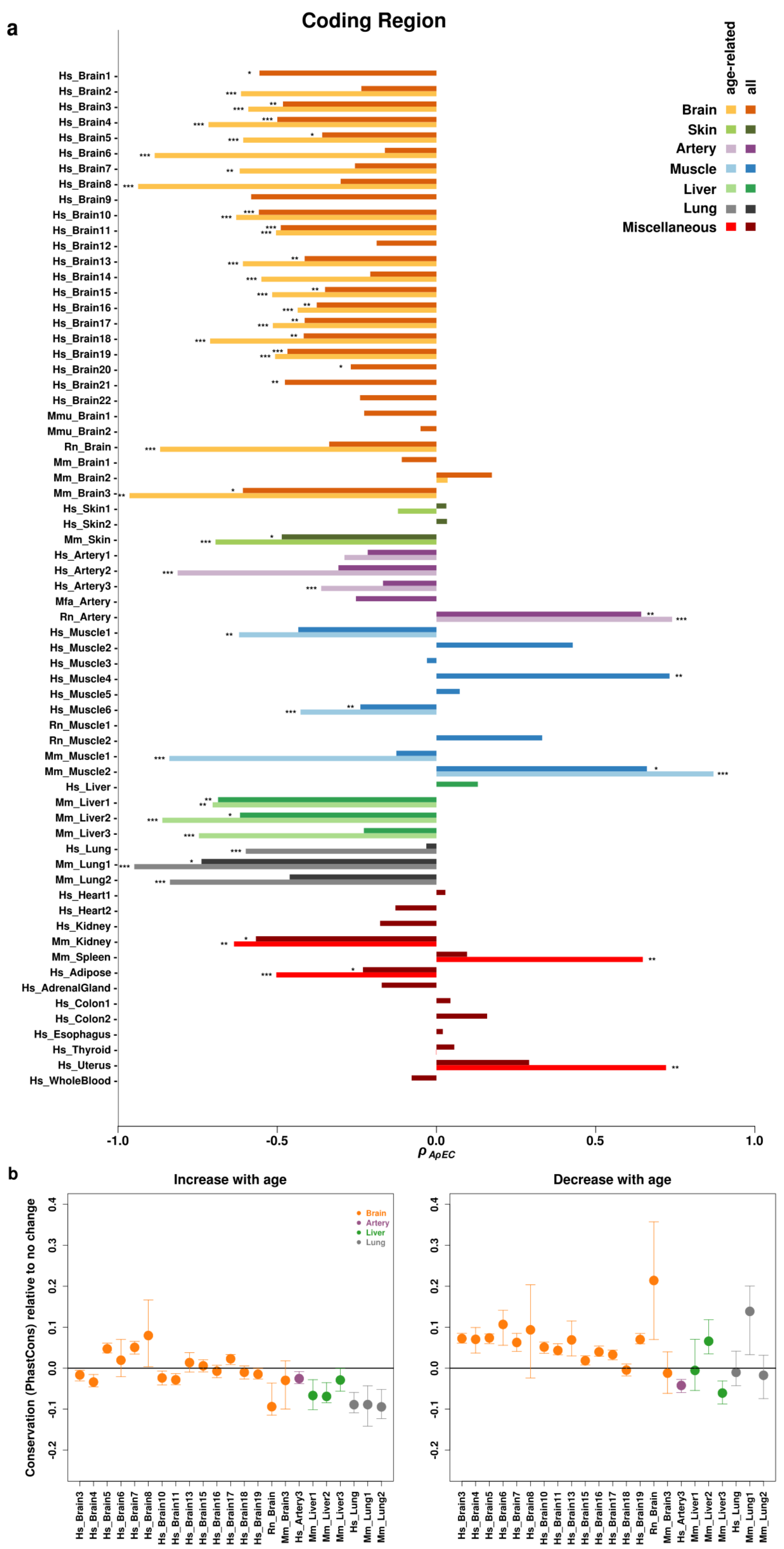

Supplementary Figure 7. Age-related changes in conservation of coding regions measured using the PhastCons metric. **(a)** The x-axis shows age-dependent change in expression level–regulatory region conservation correlation, measured by the Spearman correlation coefficient ρ. The results were calculated separately for each dataset, and for significant age-related genes in that dataset (light bars), as well as for all expressed genes (dark bars). Note that in 40 of 66 datasets (cases where the light bar is missing), no significant age-related gene set could be identified at $q < 0.10$.

The asterisks indicate, (*): $p \leq 0.05$, (**): $p \leq 0.01$, (***): $p \leq 0.001$. **(b)** Comparison of the conservation metric among gene sets showing different age-related expression level change patterns. The plots show mean conservation metric for genes showing age-related increase (left) and age-related decrease (right) in expression level, compared to mean conservation metric among genes showing no significant age-related change in expression level. The error bars indicate 95% confidence intervals calculated by 1,000 bootstraps. In 13 datasets (bootstrap support >95%), genes that show an increase in expression with age had lower regulatory region conservation, on average, than genes with no change. In 15 datasets (bootstrap support >95%) genes that show a decrease in expression with age also had higher conservation levels, on average, than genes with no change.

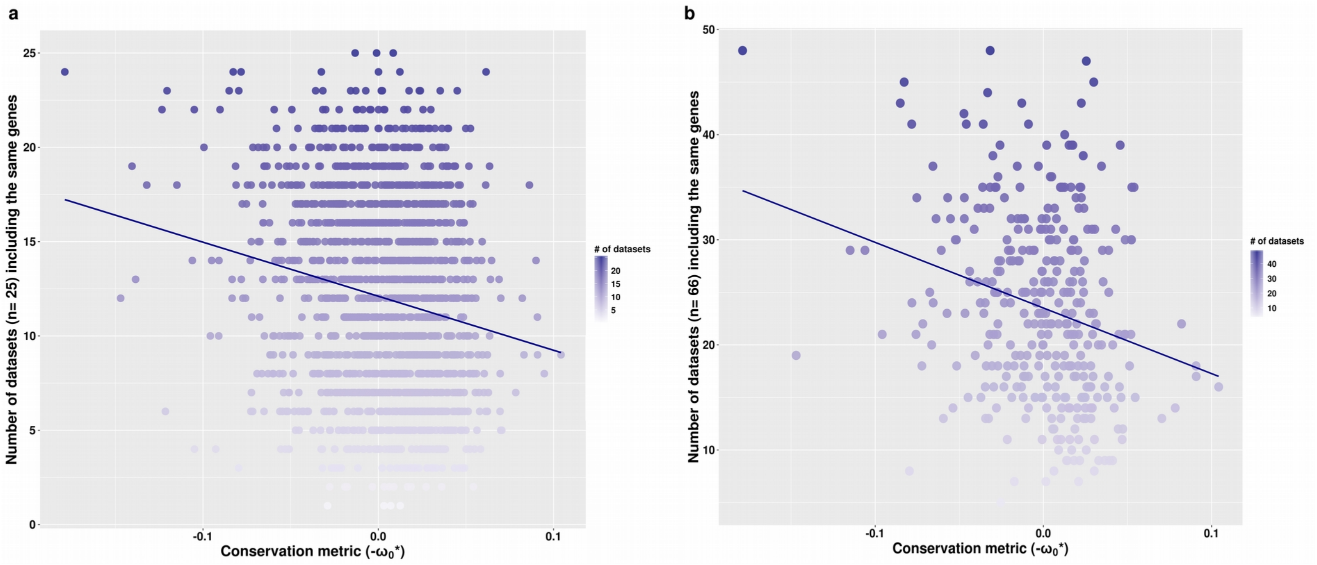

Supplementary Figure 8. Correlation between gene protein sequence conservation metrics and the frequency of datasets where the same gene shows increase in expression with age. The x-axes and y-axes show conservation ($-\omega_0^*$) and number of datasets in which a gene shows $\rho_{EA} > 0$ among **(a)** the 25 ADICT-associated datasets and **(b)** all 66 datasets, respectively. Darker colour indicates higher rates of gene sharing between datasets. Spearman correlation coefficient rho is -0.17 ($p < 0.001$) for panel (a) and -0.23 ($p < 0.001$) for panel (b). Note that genes in panel (a) will not be represented in panel (b) if they are not detected in some of the 41 datasets not showing ADICT.

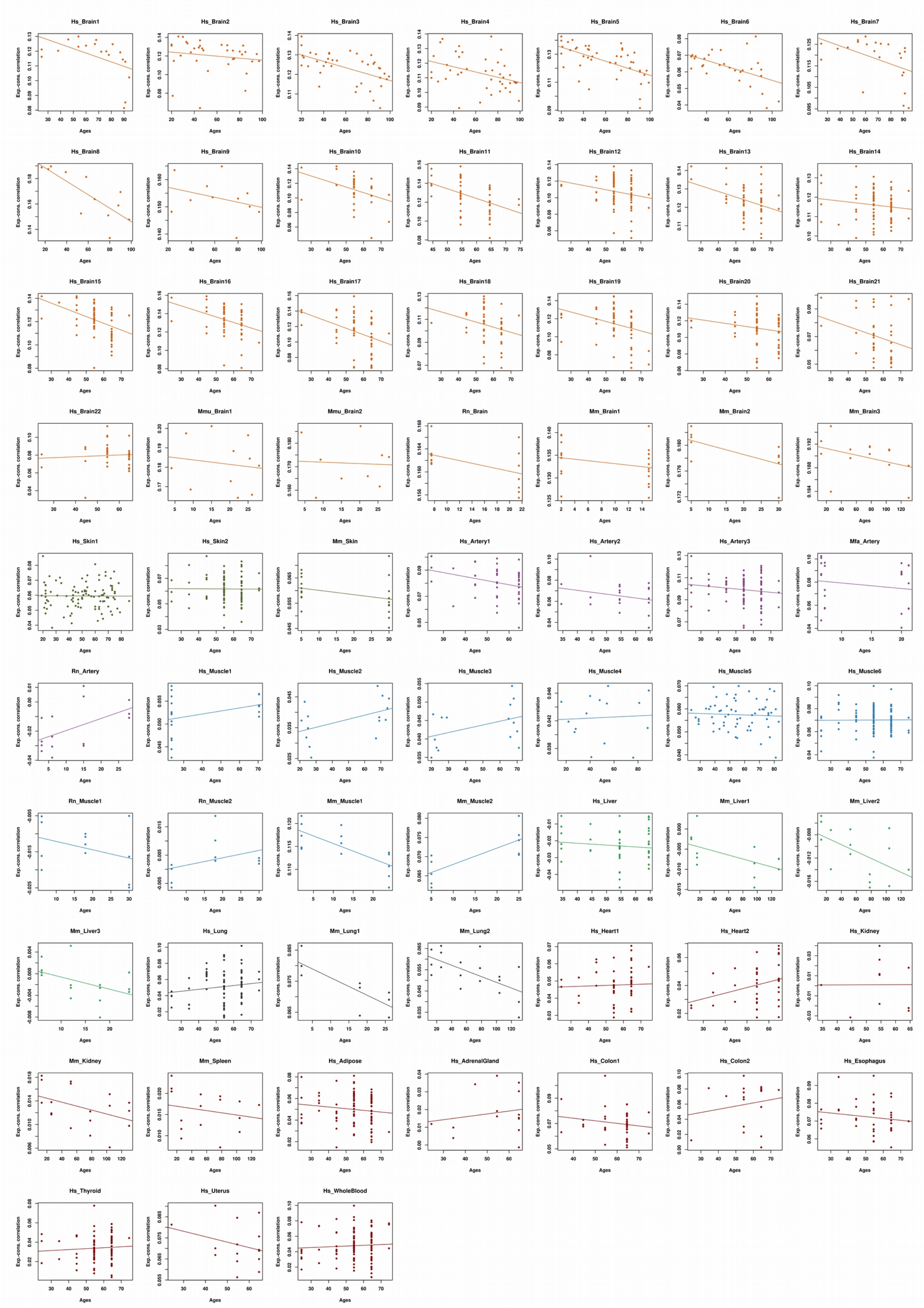

Supplementary Figure 9. Changes in the correlation between expression and conservation metric (-ω0*) with age across all 66 datasets, calculated using all expressed genes. The correlations are those represented in Fig. 2 and in Supplementary Table 2. Our aim here was to understand whether

correlation analysis would be appropriate for studying the expression-conservation correlation and age relationship. Indeed we fnd that, for datasets showing ADICT trend, a linear model fits the data better ($p < 0.05$) than an alternative quadratic model in 76% of cases (calculated using the R function “lm”).

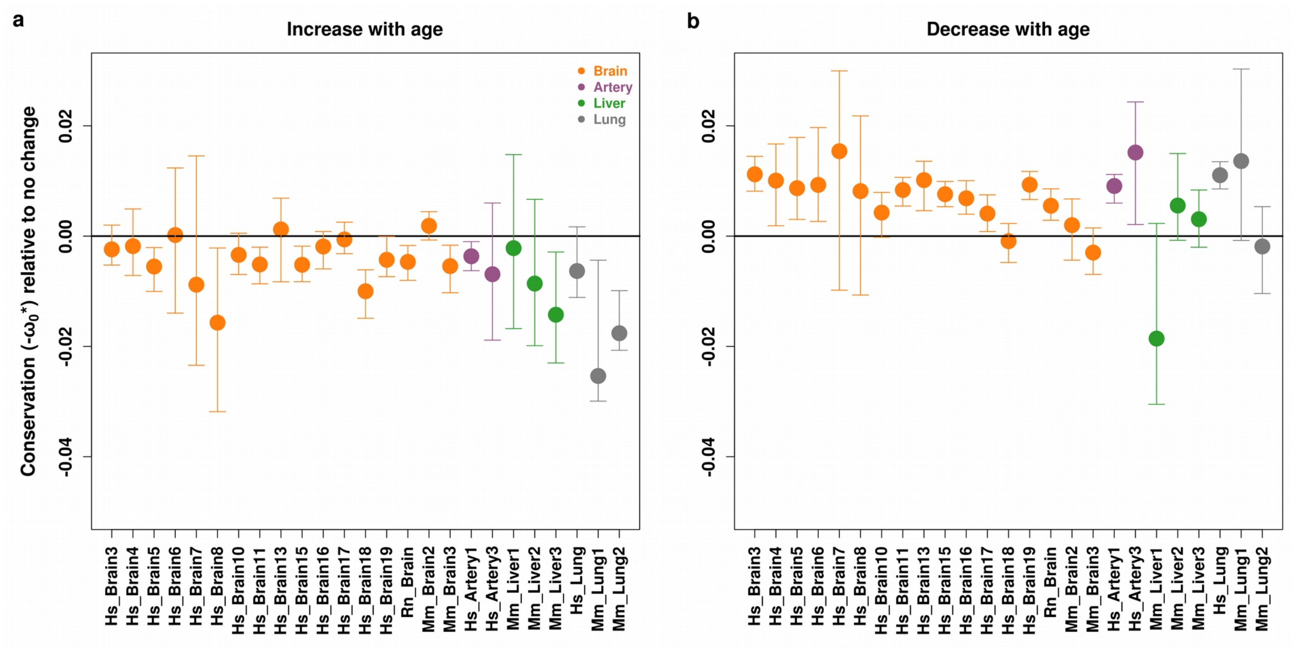

Supplementary Figure 10. Comparison of conservation metric among gene sets that show different age-related changes in expression, after removing immune system related genes. The plots show mean conservation metric for genes showing age-related increase (left) and age-related decrease (right) in expression level, compared to mean conservation metric among genes showing no significant age-related change in expression level. The error bars indicate 95% confidence intervals calculated by 1,000 bootstraps. In 12 datasets (bootstrap support >95%), genes that show increases in expression with age had on average lower regulatory region conservation, and in 15 (bootstrap support >95%) of these datasets, genes that show decreases in expression with age also had on average higher conservation than genes with no change.

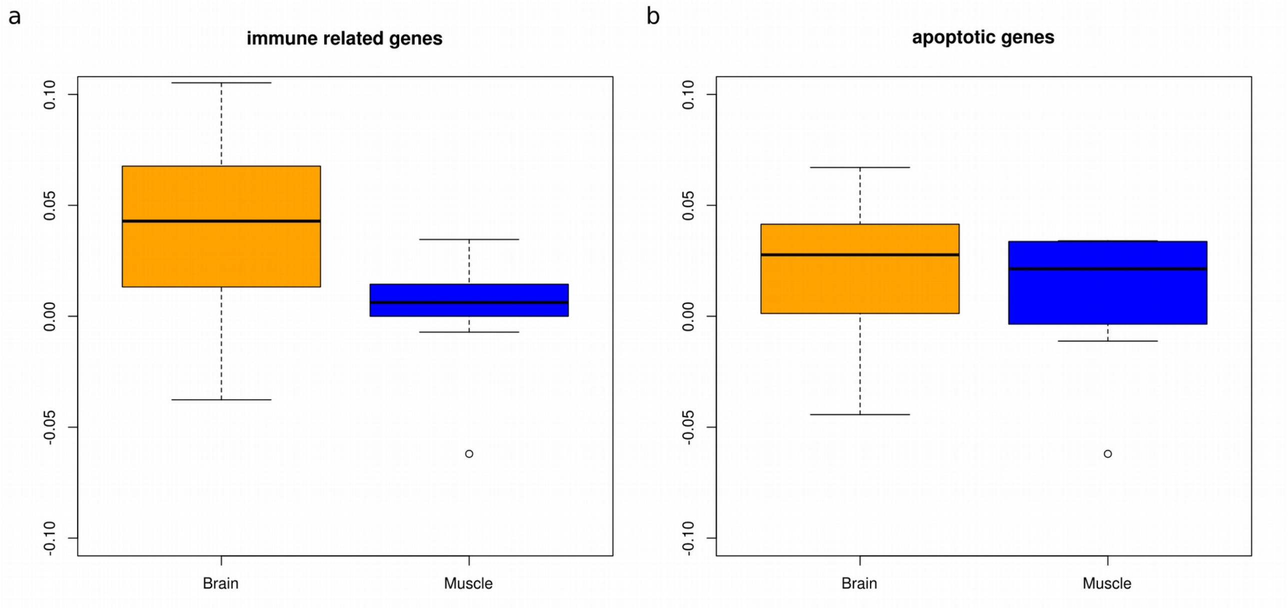

Supplementary Figure 11. Age-related increases in expression differ between brain and muscle. The y-axis shows the average expression-age correlation coefficient (rho) calculated across datasets of a specific tissue. (a) Genes (n=3171) in immune-related pathways (a group enriched among IELC genes compared to other genes) are more strongly up-regulated with age in brain (an ADICT tissue) than muscle (a non-ADICT tissue) (Mann-Whitney U test p = 0.006). (b) Apoptosis-related genes (n=1913) show a similar but weak trend, which is not significant (Mann-Whitney U test p = 0.39). Note that these functional groups have been identified as being enriched among IELC genes across ADICT tissues (i.e. genes showing consistent increase in expression with age and low evolutionary conservation), but not as being differently expressed between ADICT and non-ADICT tissues.

Supplementary Tables

Supplementary Table 1: Information about the 66 datasets used in the analysis.

Supplementary Table 2: Age-dependent changes in transcriptome conservation ($\rho_{A\rho EC}$) calculated for different conservation metrics and gene sets. **(a)** The number of age-related and all expressed genes calculated for the 66 datasets. Sheets B-F contain results (number of genes, $\rho_{A\rho EC}$, *p*-values) calculated using **(b)** $-\omega_0$ as conservation metric; **(c)** $-\omega_0^*$ as conservation metric (the main result in our analyses), **(d)** $-\omega$ (or *dN/dS*) for "one-to-one orthologs" between human-mouse as conservation metric, **(e)** $-\omega$ for "one-to-one orthologs" between human-elephant as conservation metric, **(f)** $-\omega$ for "one-to-one orthologs" between human-cow as conservation metric. Sheets G-I contain results (number of genes, $\rho_{A\rho EC}$, *p*-values) calculated using gene sets excluding putatively positively selected genes (with $\omega > 1$ in our data), immune system genes, and down-regulated genes in each dataset (as indicated with the "WID" suffix). We repeated this analysis using **(g)** $-\omega$ (or *dN/dS*) for "one-to-one orthologs" between human-mouse as conservation metric, **(h)** $-\omega$ for "one-to-one orthologs" between human-elephant as conservation metric, **(i)** $-\omega$ for "one-to-one orthologs" between human-cow as conservation metric. All results are calculated for all genes and age-related genes in a dataset.

Supplementary Table 3: Results of the comparison of the linear and quadratic regression models in 25 datasets that show an ADICT trend. A significant *p*-value indicates a better fit of the linear model, estimated by the R "lm" function.

Supplementary Table 4: Results of REVIGO analyses.
